# Topical Gentamicin in the Management of Bart Syndrome: A Case Report

**DOI:** 10.7759/cureus.104130

**Published:** 2026-02-23

**Authors:** Bouchra El Ghouti, Marwa Faik Ouahab, Soumiya Chiheb, Madiha Eljazouly

**Affiliations:** 1 Dermatology Unit, Cheikh Khalifa International University Hospital, Casablanca, MAR; 2 Dermatology Unit, Ibn Rochd University Hospital, Casablanca, MAR; 3 Dermatology Unit, Cheikh Khalifa International University Hospital, Mohammed VI University of Health Sciences, Casablanca, MAR

**Keywords:** aplasia cutis congenita, bart syndrome, bullous dermatoses, inherited epidermolysis bullosa, topical gentamicin

## Abstract

Bart syndrome is a rare congenital disorder characterized by localized absence of skin, epidermolysis bullosa (EB), and nail abnormalities. We report the case of a female newborn presenting with congenital absence of skin on both feet, associated to multiple blisters in friction-prone areas and paronychia. Despite these findings, she remained clinically stable, was feeding normally, and exhibited no gastrointestinal symptoms. Based on the association of aplasia cutis and epidermolysis bullosa, a diagnosis of Bart syndrome was clinically suspected. The patient was treated with topical gentamicin 0.5% diluted in petroleum jelly, applied twice weekly, combined with a non-adhesive dressing. Clinical improvement was observed, with complete healing of the perineal lesions within three months and significant regression of limb involvement. Although spontaneous re-epithelialization is part of the natural course of the disease, the rapid and sustained clinical evolution raises the possibility of a beneficial effect of gentamicin. To the best of our knowledge, similar responses in this specific context have rarely been reported. Larger studies are required to confirm efficacy, define optimal treatment protocols, and evaluate long-term safety.

## Introduction

Bart syndrome, first described by Bart in 1966, is a rare congenital disorder characterized by aplasia, congenital epidermolysis bullosa (EB), and associated nail abnormalities [1]. It is currently considered a clinical phenotype within the spectrum of epidermolysis bullosa (EB), rather than a distinct nosological entity, most frequently associated with dystrophic forms of EB [2].

Epidermolysis bullosa is a group of inherited disorders caused by mutations in structural proteins responsible for dermo-epidermal adhesion. In dystrophic EB, mutations in COL7A1 result in defective type VII collagen, leading to skin fragility and blistering after minor trauma [2].

The disease typically manifests at birth with localized absence of skin followed by blister formation in trauma-prone areas. Although precise epidemiological data are lacking, Bart syndrome represents an exceptionally rare presentation within the already uncommon spectrum of EB [2]. Clinical severity varies widely, but management remains largely supportive, relying on careful wound care, infection prevention, and mechanical protection of the skin [3]. To date, no curative therapy is available, highlighting an important unmet therapeutic need.

In recent years, increasing interest has emerged in therapeutic strategies targeting the molecular basis of inherited skin fragility disorders. Among these, aminoglycosides such as gentamicin have been investigated for their potential role in restoring functional protein synthesis in selected genetic contexts. In the presence of nonsense mutations, premature stop codons truncate protein synthesis. Aminoglycosides may induce read-through of premature termination codons, allowing partial restoration of type VII collagen synthesis in patients harboring nonsense mutations [4]. However, evidence regarding their benefit in Bart syndrome remains extremely limited.

We report a neonatal case with a favorable clinical evolution following topical gentamicin therapy. To the best of our knowledge, similar outcomes in this context have rarely been documented, and the present case highlights a favorable clinical response in this setting.

## Case presentation

A female newborn, the third child of a consanguineous marriage, was delivered via cesarean section at 38 weeks of gestation and referred to the neonatal intensive care unit because of extensive congenital skin lesions. Both parents were in good health, with no abnormalities of the skin, skin appendages, or mucous membranes. Notably, a cousin had also been affected by a similar condition, as revealed through family history and parental recollection, and had unfortunately passed away due to complications of this disease.

Clinical examination revealed well-demarcated areas of congenital skin absence involving the dorsal and plantar aspects of both feet, with partial dermal exposure revealing an erythematous, vascularized base. The lesions had sharp borders separating affected and unaffected skin, without signs of infection or necrosis, consistent with aplasia cutis.

**Figure 1:**
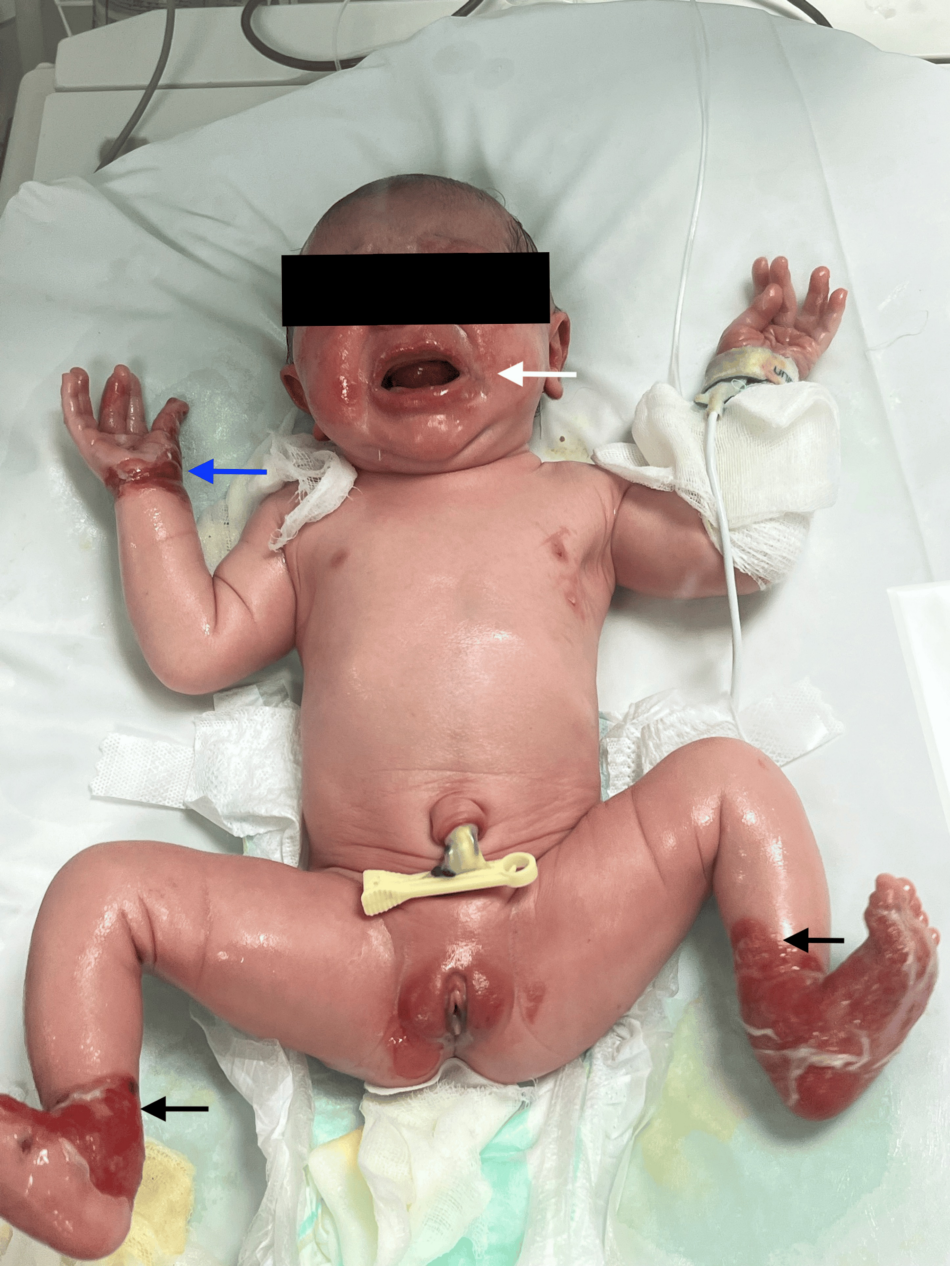
Clinical presentation at birth showing congenital absence of skin involving the distal lower extremities (black arrows) with sharply demarcated borders, associated with erosions and blistering in friction-prone areas (blue arrow). Perioral involvement is also visible (white arrow).

**Figure 2:**
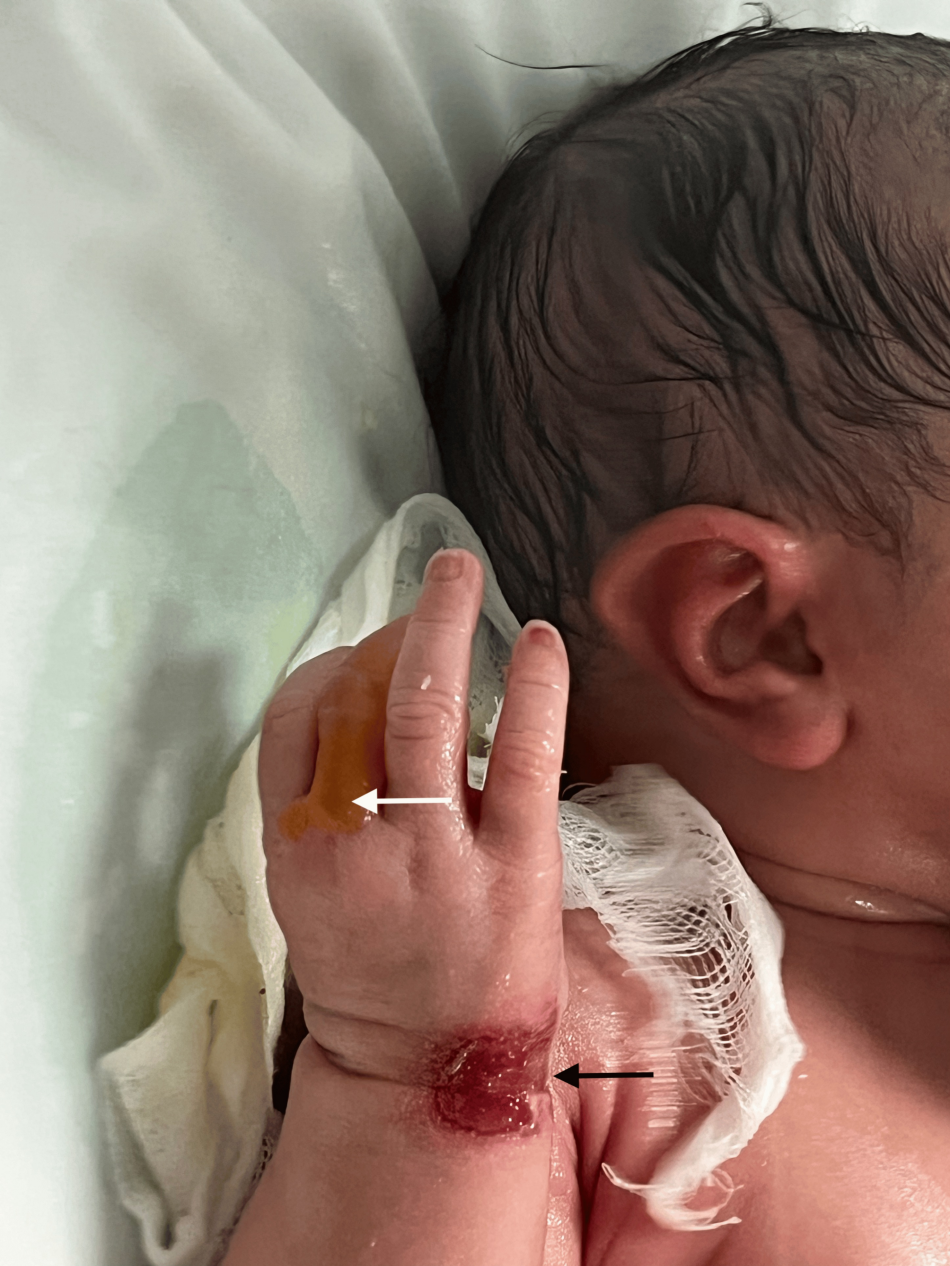
Close-up view at birth of the hand and wrist showing a well-defined erosion at the wrist (black arrow) and a blister on the finger (white arrow), illustrating skin fragility in trauma-prone areas.

Additionally, the newborn developed blisters in friction-prone areas, including the limbs, wrists, fingers, perineal region, and oral mucosa (Figure 1 and Figure 2).

Moreover, the patient presented dystrophic nail changes associated with paronychia. Despite these findings, she was otherwise clinically stable and feeding normally, without vomiting or abdominal distention.

Given the association of aplasia cutis and trauma-induced blistering, mucosal involvement, nail abnormalities, and a suggested family history in a consanguineous context, a clinical diagnosis of Bart syndrome was strongly suspected. Differential diagnoses such as isolated aplasia cutis congenita, junctional epidermolysis bullosa, and infectious bullous disorders were less likely based on the distribution of lesions, trauma-induced blistering, and absence of systemic signs of infection.

However, a skin biopsy and genetic testing were not performed due to parental refusal, which represents a limitation in definitive molecular classification.

**Figure 3:**
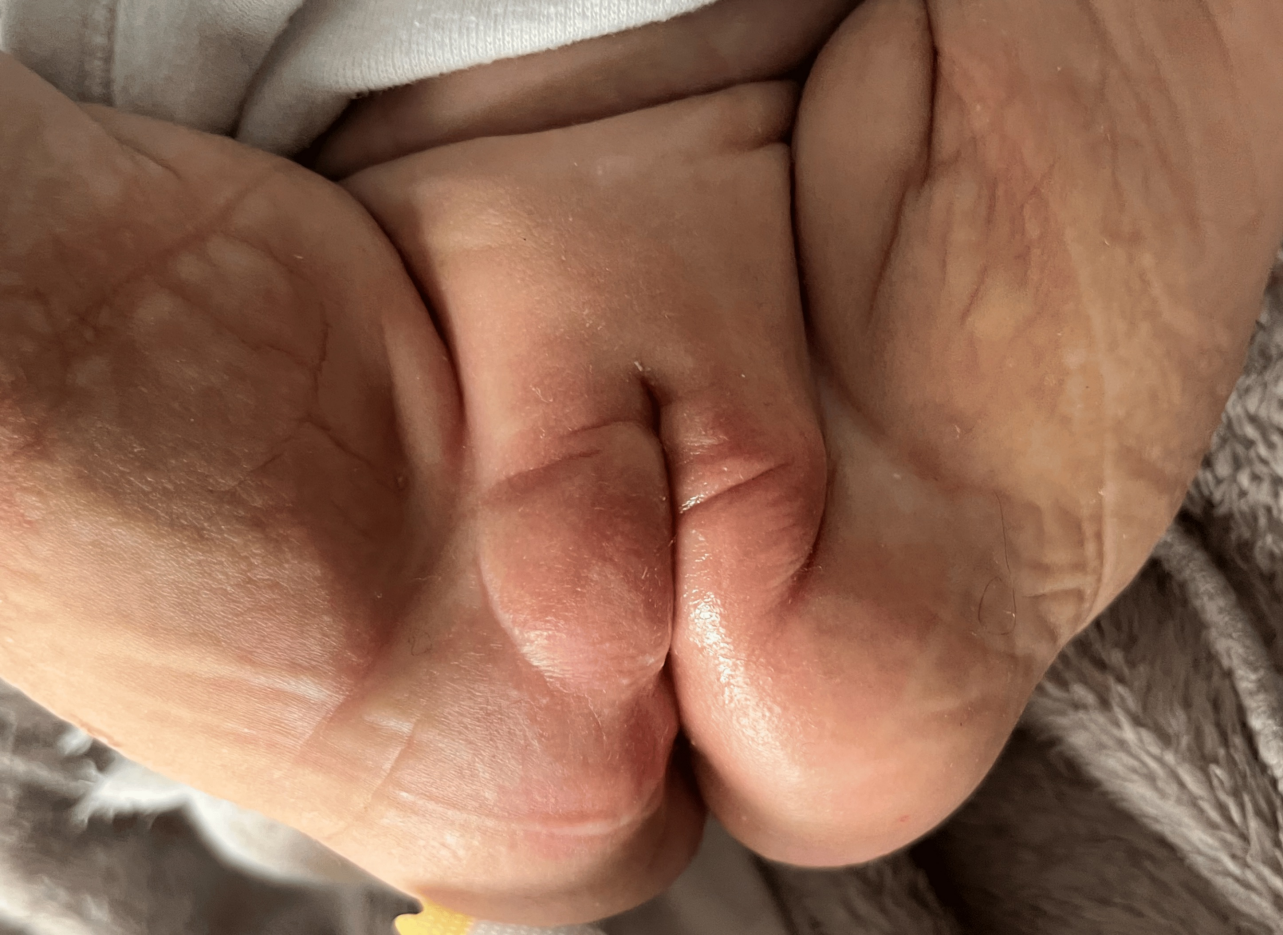
Follow-up at three months demonstrating healed skin with no residual erosions or blisters.

**Figure 4:**
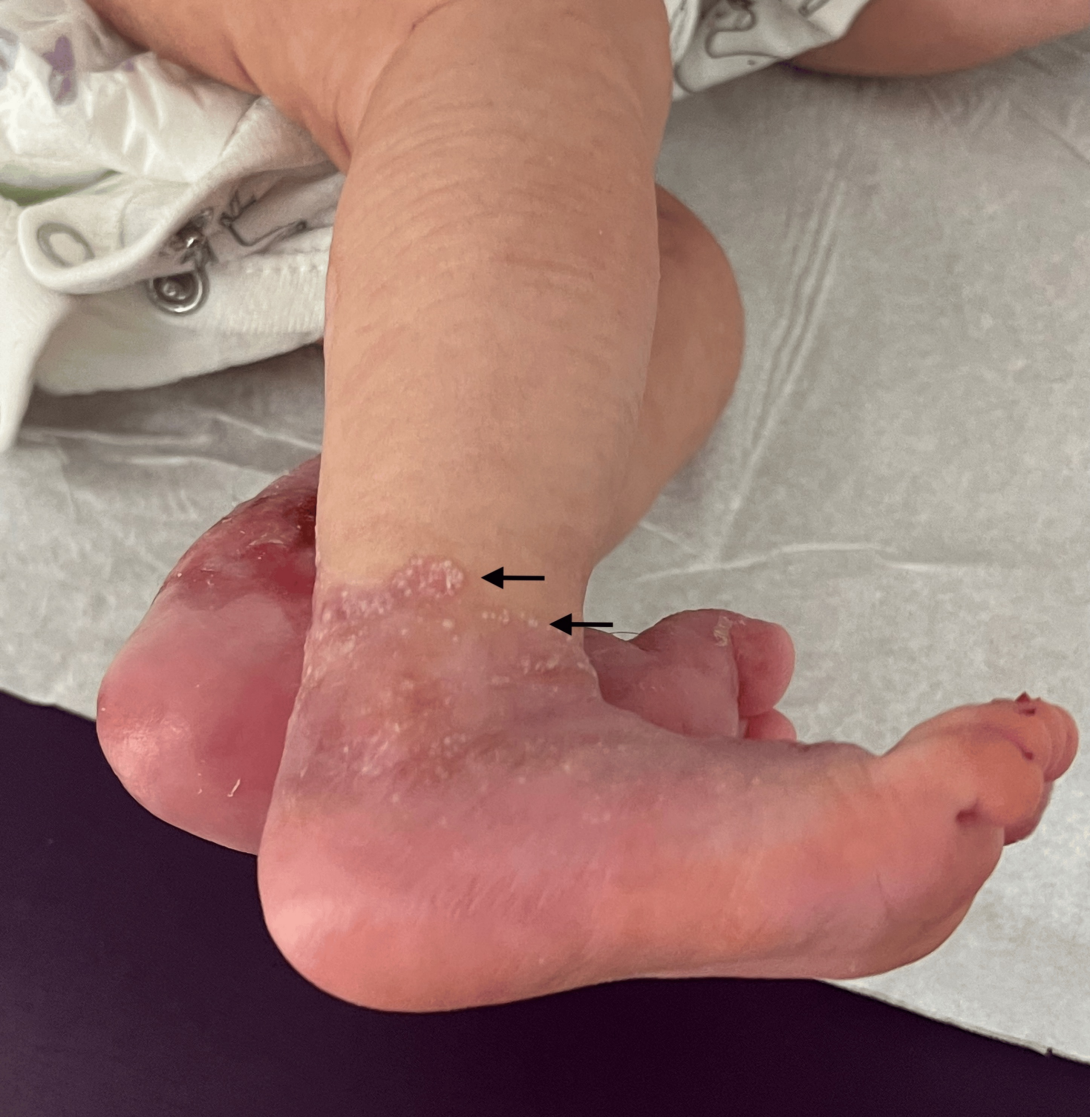
Clinical evolution after three months of treatment showing re-epithelialization of previously involved areas, with secondary milia formation (black arrows).

Treatment was initiated with topical gentamicin 0.5% diluted in petroleum jelly twice weekly, and a non-adhesive dressing to promote healing. At three months follow-up, complete healing of blisters on the perineal region and face was noted (Figure 3), along with the progressive regression of limb lesions with secondary milia formation observed at the periphery of previously affected areas (Figure 4), without new blister formation in healed areas.

## Discussion

Bart syndrome is an autosomal dominant disorder, although sporadic cases occur. It manifests as well-defined, shiny red lesions, often extending from the feet to the thighs. While any skin region can be affected, lesions predominantly appear in high-friction areas such as the hands, feet, limbs, and perioral skin [5]. The gastrointestinal tract, ocular, and genitourinary mucosa may also be involved [6]. Furthermore, while the coexistence of Bart syndrome and congenital pyloric atresia has been documented, it remains rare [7]. In our patient, no extracutaneous involvement was identified.

The term Bart syndrome is used to describe patients with any form of EB who present with localized congenital absence of skin on the extremities [1]. Accurate classification of the EB subtype is crucial for prognosis and genetic counseling [6]. Notably, Bart syndrome is generally associated with a more favorable prognosis than other EB subtypes [8].

The proper diagnosis of this syndrome is established by genetic testing and sometimes by antigen mapping and histopathological analysis [9].

An analysis of the Bart syndrome family of origin linked the inheritance of the disease to a region of chromosome 3 close to the type VII collagen gene (COL7A1) [10]. The condition is inherited in an autosomal dominant manner, although sporadic mutations involving COL7A1 may be seen [11].

Management is primarily conservative, focusing on wound care, infection control, and protective dressings such as petrolatum gauze. Blisters can be drained, and topical agents such as silver sulfadiazine may aid healing. Systemic antibiotics are used only for infections, while severe cases may require surgical intervention, including skin grafting or local flaps [12]. The aplasia cutis usually re-epithelializes within 6-8 weeks of proper care, with granulation and scarring, as observed in our patient, while EB stays a lifelong issue [11].

Some authors have documented the efficacy and safety of topical gentamicin diluted between 0.1% and 0.5% in petroleum jelly [13] or a collagen base [14] for healing lesions in dominant dystrophic epidermolysis bullosa (DDEB).

Aminoglycosides are a class of antibacterial agents that promote the read-through of premature stop codons, thanks to the induction of a tRNA during translation, helping the production of a functional collagen VII instead of a truncated protein. Studies have also shown promising results in other diseases linked to this type of mutation, such as junctional EB, Duchenne muscular dystrophy, and hemophilia [14].

Although the use of topical gentamicin in Bart syndrome has not been specifically detailed in previous publications, several reports have highlighted the benefit of aminoglycoside-induced read-through therapy in other COL7A1-related disorders.

In patients with recessive dystrophic EB carrying nonsense mutations, topical or locally injected gentamicin has been associated with enhanced type VII collagen expression, improved anchoring fibril formation, and acceleration of wound closure [15]. Additional case series have described partial restoration of collagen VII at the dermo-epidermal junction, together with a reduction in blister formation [16].

However, not all studies have demonstrated uniform efficacy, as some cohorts treated with less intensive regimens failed to show significant clinical improvement [17].

Compared with previously published reports, the clinical evolution observed in our patient appeared temporally favorable. The relatively rapid re-epithelialization and stability of skin integrity during follow-up may raise a hypothesis regarding a potential therapeutic contribution; however, causality cannot be established in the absence of molecular confirmation and controlled comparison. While this observation may justify further investigation into aminoglycoside-based read-through strategies in selected cases of Bart syndrome [18], it does not establish therapeutic efficacy [18].

Nevertheless, several limitations must be acknowledged. The absence of molecular confirmation of a nonsense mutation prevents definitive attribution of the clinical response to aminoglycoside-induced read-through. In addition, spontaneous improvement of aplasia cutis is part of the natural history of the disease and may partly confound the interpretation of treatment efficacy. Despite these reservations, the favorable tolerance, accessibility, and low cost of topical gentamicin make it an attractive adjunctive option while awaiting stronger evidence.

## Conclusions

Topical gentamicin use in our patient was temporally associated with rapid improvement of erosions and restoration of skin continuity, with good tolerance. While spontaneous healing cannot be excluded, and given the absence of molecular confirmation, this observation should be considered hypothesis-generating rather than evidence of efficacy. This single observation does not establish therapeutic efficacy but may generate hypotheses for future evaluation of amino glycoside-based read-through strategies in selected cases of Bart syndrome. Further prospective studies will be necessary to confirm efficacy, define optimal treatment protocols, and evaluate long-term outcomes.
